# Transient colonizing microbes promote gut dysbiosis and functional impairment

**DOI:** 10.1038/s41522-024-00561-1

**Published:** 2024-09-08

**Authors:** Sunjae Lee, Victoria Meslier, Gholamreza Bidkhori, Fernando Garcia-Guevara, Lucie Etienne-Mesmin, Frederick Clasen, Junseok Park, Florian Plaza Oñate, Haizhuang Cai, Emmanuelle Le Chatelier, Nicolas Pons, Marcela Pereira, Maike Seifert, Fredrik Boulund, Lars Engstrand, Doheon Lee, Gordon Proctor, Adil Mardinoglu, Stéphanie Blanquet-Diot, David Moyes, Mathieu Almeida, S. Dusko Ehrlich, Mathias Uhlen, Saeed Shoaie

**Affiliations:** 1 Centre for Host-Microbiome Interactions, Faculty of Dentistry, Oral & Craniofacial Sciences, King’s College London, London, SE1 9RT UK; 2 School of Life Sciences, Gwangju Institute of Science and Technology, Jouy-en-Josas, 61005 Republic of Korea; 3 University Paris-Saclay, INRAE, MetaGenoPolis, 78350 Jouy-en-Josas, France; 4 Science for Life Laboratory, KTH – Royal Institute of Technology, Stockholm, SE-171 21 Sweden; 5 Université Clermont Auvergne, INRAE, UMR 454 MEDIS, 28 place Henri Dunant, F-63000 Clermont-Ferrand, France; 6 Department of Bio and Brain Engineering, KAIST, 291 Daehak-ro, Yuseong-gu, Daejeon, 305-701 Republic of Korea; 7 Centre for Translational Microbiome Research, Department of Microbiology, Tumour and Cell Biology, Karolinska Institutet, Stockholm, SE-171 77 Sweden

**Keywords:** Microbiome, Next-generation sequencing, Metagenomics, Microbial ecology

## Abstract

Species composition of the healthy adult gut microbiota tends to be stable over time. Destabilization of the gut microbiome under the influence of different factors is the main driver of the microbial dysbiosis and subsequent impacts on host physiology. Here, we used metagenomics data from a Swedish longitudinal cohort, to determine the stability of the gut microbiome and uncovered two distinct microbial species groups; persistent colonizing species (PCS) and transient colonizing species (TCS). We validated the continuation of this grouping, generating gut metagenomics data for additional time points from the same Swedish cohort. We evaluated the existence of PCS/TCS across different geographical regions and observed they are globally conserved features. To characterize PCS/TCS phenotypes, we performed bioreactor fermentation with faecal samples and metabolic modeling. Finally, using chronic disease gut metagenome and other multi-omics data, we identified roles of TCS in microbial dysbiosis and link with abnormal changes to host physiology.

## Introduction

The human gut microbiota is a microbial community continuously colonizing the host from early life. It is established over the lifespan of the host and changes over time. Stability of the gut microbial ecosystem is associated with a high degree of microbial richness and a consecutive diverse functionality. This, in turn, confers a high degree of metabolic plasticity allowing for the microbial production of a wide range of fermented metabolites with various beneficial effects on the host^1,2^. Although the healthy adult gut microbiome composition is stable and resilient, it can experience periodic or continuous perturbations driven by external factors that shift the microbial colonization profile to a transient state that can either undergo reversion to the initial profile or progression to a new stable community. In doing so, there may be changes in functional redundancy and transition to alternative, possibly dysbiotic communities which can then result in changes in host physiology^3^.

Understanding this transition and the impacts on the host requires longitudinal sampling of gut microbiota of large populations together with observations of changes in host physiology. Previously, several studies have investigated gut microbial taxa and compositional adaptations in early life and childhood^4–8^. Although gut microbiome longitudinal studies have revealed changes in the metagenome taxa over time and associated functional shifts^9^, the ecological destabilization of the core gut microbiota, notably the underlying species and their functions, has been far less investigated. This might provide a new perspective of understanding key community structure of human gut microbiome, which cannot be captured by the core microbiota defined by the sample frequency of cross-sectional study.

Here, we modeled microbial persistence in the human gut microbiota using data from a longitudinal cohort from the healthy Swedish population over a one-year period, identifying two distinct microbial populations – persistent colonizing species (PCS) and transient colonizing species (TCS). Interestingly, we found that PCS fostered richness, whereas TCS destabilized the microbial community, such destabilization, known as Anna Karenina Principle (AKP) effect^10^, likely leading to dysbiotic microbiome. We further validated this species classification by expanding the longitudinal data by including two additional time points over six months and comparing this data set with two independent longitudinal gut microbiome published data sets from different geographical regions. These analyses revealed that TCS rarely grow well in the host, but they increase in disease populations and/or where a dysbiotic microbiome is developing. An in-depth functional study of PCS and TCS will therefore uncover new physiological roles of human gut microbiota in health and disease, identifying potential pathological mechanisms for future therapeutic intervention.

## Results

### Two distinct microbial populations with varying microbial persistence identified using probabilistic model

**Fig. 1:**
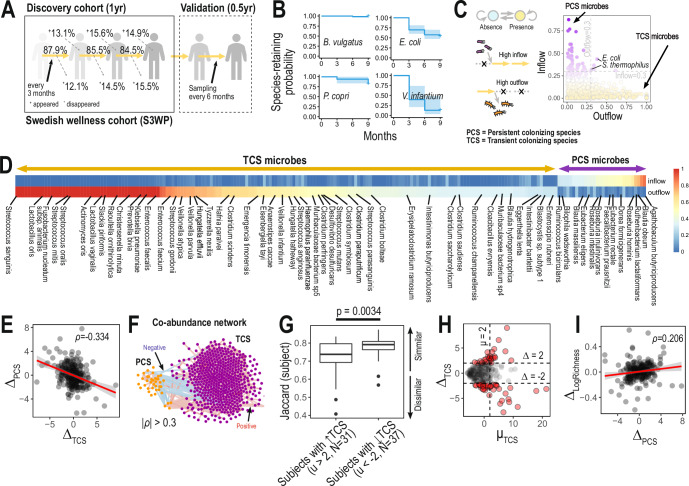
Persistent and transient colonizing species determine the stability of the gut microbiota. **A** Characterization of the temporal changes in the gut microbiota in 86 Swedish healthy individuals (Swedish wellness cohort, S3WP) over the course of a year (total of 344 samples) with an estimation of the proportion of persistent species over the time points. **B** Species retention probability estimated by Kaplan–Meier statistics. Based on the presence/absence events of a given species among the four sampling points, we estimated the retention probability from Kaplan–Meier estimators. As examples, we show Kaplan–Meier plots of four different species with varying retention probabilities (e.g., *Veillonella infantium, Bacteroides vulgatus, and Prevotella copri*). **C** Modeling the temporal changes of species, referred to here as the microbial flux, by Markov chain models (MCMs). Based on the models, the species transition probabilities of consecutive sample points from absence to presence (inflow) or vice versa (outflow) were estimated (left top); dotted lines with a cross and gold arrows represent failure and success in detecting a species, respectively (left bottom). Next, inflow vs outflow score plot (right panel) identifies persistence colonizing species (PCS) (species with higher inflow (>0.3) and lower outflow (<0.3); e.g., *Agathobaculum butyriciproducens* and *Blautia obeum*), transient colonizing species (TCS) (species with lower inflow (<0.3) and higher outflow ( > 0.3); e.g., *Veillonella parvula* and *Hungatella effluvia*) or species that colonizes stochastically (e.g., *Escherichia coli* and *Streptococcus salivarius*). **D** Inflow and outflow scores of PCS and TCS species, together with taxon (those with unclassified taxa not shown). **E** Negative correlation of abundance changes between persistent colonizing species (∆_PCS_) and transient colonizing species (∆_TCS_) between consecutive sample points (spearman’s *ρ* = −0.334, *p*-value < 0.05). **F** Co-abundance network analysis identified negative correlations of abundances between PCS and TCS microbes (blue and red edges represented negative and positive Spearman’s correlation coefficients, respectively). **G** Decreased intra-individual Jaccard similarity of TCS-enriched individuals. We identified TCS-enriched individuals among 86 healthy individuals based on scaled total abundance of TCS species by Z-score (Z_t_ and Z_t+1_) and its mean between sample points (µ_TCS_ = ½ × (Z_t_ + Z_t+1_) > 2). TCS-enriched individuals were less similar between visits than TCS-depleted individuals (Student’s *t* test *p*-value < 0.01). **H** Abundance changes of TCS species between consecutive sample points (∆_TCS_) over the mean value (μ_TCS_). Unlike PCS species, the abundance of TCS species substantially changed between consecutive sample points (|∆_TCS_|< 2) according to the increase in mean values (μ_TCS_). **I** Correlation between changes in PCS species abundance (∆_PCS_) and richness changes (spearman’s ρ = 0.206, *p*-value < 0.05). For boxplots, Q1, median, and Q3 quantiles of given boxes were denoted, together with outliers shown as dots.

In order to explore the stability of the human gut microbiome, we set out to investigate the temporal changes in the human gut microbiota composition of 86 healthy Swedish individuals (Supplementary Table 1), at four time points over a one-year period, sampling every three months (Fig. 1A). To this end, we generated whole-genome deep sequenced shotgun metagenomics data of participants’ stool samples (30 million reads on average) (Methods). The non-redundant integrated gene catalog for human gut microbiome (IGC2)^11^ was used to generate the gene counts with rarefaction of the aligned reads into 10 million reads to correct the biases of different sequencing depths (Methods). Using metagenomic species (MGS) as a reference to identify the gene clusters representing microbial species, we profiled the microbial populations of these samples at the MGS level detecting 1413 microbial species (out of a possible total of 1989 MGS in the catalog) in our longitudinal cohort (Methods).

We compared the number of detected MGS for individuals at different time points, demonstrating around 86% of species were found to be shared between consecutive visits (Fig. 1A). In addition, across all 86 individuals, we traced the retention periods of individual MGS using Kaplan–Meier estimates, referred to here as the species retention probability (Fig. 1B and Methods). Interestingly, we observed different ranges of retention probability among detected MGS. For example, *Bacteroides vulgatus* and *Prevotella copri*, well-known gut commensals, had the highest retention probability, whereas some pathobionts or microbes more usually derived from different origins than the gut such as oral cavity (e.g., *Veillonella infantium*) had reduced retention probabilities. These species retention probabilities were correlated with species mean abundance (Supplementary Fig. 1A, B), but associations did not appear significant for any individual species based on Cox regression (*p*-values > 0.1, Supplementary Fig. 1C) (For more detailed information of longitudinal metagenome data – Supplementary Fig. 5).

Next, we investigated the microbial persistence turnover of the human gastrointestinal (GI) tract (referred to hereafter as the microbial flux**)** by applying Markov chain models (MCMs) to the MGSs identified in our current cohort (Methods). This analysis enabled us to estimate the transition probability of individual species from presence to absence (outflow probability) and vice versa (inflow probability) across different sampling times. We identified two groups of species preferably transiting from presence to absence or from absence to presence, thereby transient or persistent colonizing in the GI tract; for brevity, we term them “transient colonizing species (TCS)” and “persistent colonizing species (PCS)”, respectively (Fig. 1C, D and Supplementary Table 2). Clearly, declaring a species absent or present depends on the detection threshold, which is in turn determined by sequencing depth. We performed the analysis at three sequencing depth levels of 5, 10, and 15 million reads, and observed largely concordant results (Supplementary Fig. 2). For instance, 35 PCS (90%) were detected at both 10 and 15 million reads levels, whilst 4 and 6 species were detected only at former and latter, respectively. Similar results were observed for TCS: 447 (88%) were detected at both levels, while 62 and 27 species were detected at 10 and 15 million reads only. Overall, both PCS and TCS probabilities were highly correlated at the three different depth levels, with a slight reduction for TCS at 5 million reads (Supplementary Table 2). To better confirm our findings of absence or presence of given species, we checked strain-resolution profiles for some representative species to check if PCS and TCS species retained similar strain also. We estimated relative abundance profiles of *Dorea formicigenerans* (PCS species) and *Raoultella ornithinolytica* (TCS species), which has substantial number of known strain reference genomes. Based on most closely related strain genomes of those two species, we checked strain abundance profiles by StrainGE tool^12^, which deconvolves strain mixtures using single nucleotide variant information, and found that *Dorea formicigenerans*, PCS species, shared similar strain profiles between consecutive visits, assuring high persistence of PCS species (Supplementary Fig. 3). However, TCS species did not share similar strain profiles, implying that TCS species tried spontaneous colonization, but failed to keep same strains to be colonized between consecutive visits.

It is important to know whether there is any link between these two groups of microbial species, so next we set out to determine whether there were any correlations between TCS and PCS populations. Interestingly, we observed that the changes of total TCS abundances by time among 86 individuals (Δ_TCS_) were inversely correlated with those of total PCS (Δ_PCS_), implying possible negative inter-bacterial interactions between PCS and TCS populations (Spearman’s correlation = −0.334, *p*-value = 4.6 × 10^−8^; Fig. 1E). We also confirmed this negative correlations between TCS and PCS populations using cross-sectional datasets from current Swedish wellness cohort, UK twin cohort^13^ (Project ID: PRJEB9584), and Germany colorectal cancer cohort^14^ (Project ID: PRJEB27928) (R ≤ −0.193, *p*-values ≤ 0.005) (Supplementary Fig. 4). Further, co-abundance network analysis showed that microbial abundances of individual PCSs and TCSs were inversely correlated with each other, and clustered into their own distinct groups (Fig. 1F). Notably, however, there were a few exceptional cases where some PCS microbes, such as *Blautia obeum*, *Ruthenibacterium lactatiformans*, *Oscillibacter sp. KLE 1728*, and *Romboutsia timonensis*, were positively correlated with TCS microbes. Importantly, these particular PCS microbes formed a distinct cluster independent of the primary PCS cluster.

We next stratified individuals into TCS-enriched (μ_TCS_ > 2; *N* = 31) and -depleted (μ_TCS_ < −2; *N* = 37) groups and traced their microbiome changes between consecutive visits (Fig. 1G, H). There was a decrease in the similarity of the gut microbiome species composition between sampling timepoints in the TCS-enriched group (Wilcoxon one-sided test p-value = 0.0034; Fig. 1G), indicating a destabilization of the structure of the gut microbiome associated with increased numbers of TCS microbes. This tendency was increased with higher mean values of TCS abundances as their abundance shifts by visits (|∆| > 2) were increased (Fig. 1H). However, PCS-enriched individuals (μ_PCS_ > 2) maintained their gut microbial composition between different time points (Supplementary Fig. 5G). Notably, increasing abundance of PCS was correlated with increasing gene richness of given gut microbiota, which is known to be related to more stable microbial communities, and thus commensal conditions. This suggests that PCS may be beneficial to host health (Spearman’s correlation = 0.206, *p*-value = 9.0 × 10^−4^; Fig. 1I and Supplementary Fig. 5H).

### Functional and phenotype features increased the survival of persistent colonizing species within the host niche

**Fig. 2:**
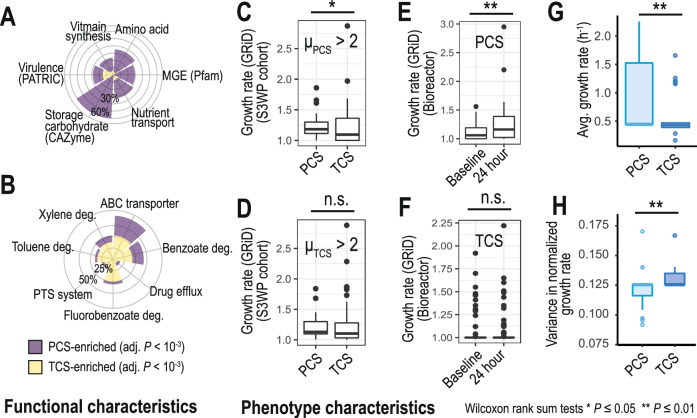
Functional and phenotype characteristics differ between persistent colonizing and transient colonizing species. Radar plots showing the fraction of biological functions or pathways enriched in either (**A**) core metabolism, virulence, or mobile genetic elements (MGEs) or (**B**) accessory metabolism, tested by linear mixed-effects models (adjusted *p*-value < 10^−3^). Persistent colonizing species (PCS) were enriched in core metabolism whilst transient colonizing species (TCS) were enriched in accessory metabolism (e.g., BTEX contaminants). We estimated physiological properties of PCS and TCS by growth rate estimations using GRiD scores (**C**–**F**) and genome-scale metabolic modeling (**G**, **H**). We estimated GRiD scores of PCS and TCS from (**C**) individuals with microbiomes highly enriched in PCS species and (**D**) individuals with microbiomes highly enriched in TCS and observed higher GRiD scores for PCS. In additional experiments investigating bioreactor fermentation of human faecal samples, we observed higher GRiD scores for (**E**) PCS after 24 h, compared to original faecal samples, whereas (**F**) TCS GRiD scores remained unchanged at 24 h. We also predicted (**G**) the growth rates and (**H**) their variance for PCS and TCS using the representative genome-scale metabolic models and found higher growth rates and less growth variances of PCS. For boxplots, Q1, median, and Q3 quantiles of given boxes were denoted, together with outliers shown as dots.

To better understand the mechanism underlying PCS and TCS survival within the host niche, we performed functional mapping of all detected species, including KEGG orthologs, PFAM protein domain, CAZyme, antiSMASH, and JGI GOLD phenotypes, and associated annotated functions to PCS and TCS species (Methods). Our functional analysis indicated that PCS were enriched in core metabolic processes, essential for energy homeostasis and for biosynthesis of macromolecules (i.e., amino acids, carbohydrates, and fatty acids; Fig. 2A, Supplementary Fig. 6 and Supplementary Tables 3, 4, Methods). They were also enriched in: (i) processes associated with increased survival, such as sporulation, cobalamin biosynthesis (CobS), and sirohydrochlorin cobaltochelatase (CbiK); (ii) secondary metabolites (bacteriocins); (iii) proteins related to starch and plant-based fiber use (CAZymes GT5, GH13, GH51); and (iv) anaerobic phenotypes (Supplementary Table 3). By contrast, TCS were enriched in accessory metabolism, such as biodegradation of xenobiotics (benzene, toluene, ethylbenzene, and xylenes - BTEX), paralleled by that of ABC transporters, possibly involved in the import of xenobiotics, suggesting that exposure to pollutants may promote their appearance (Fig. 2B and Supplementary Table 3). They were also enriched in (i) active sugar transport (i.e., phosphotransferase system (PTS); (ii) virulence factors (VFs) and trigger factors; (iii) putative competence protein ComGF and type IV secretion systems, the latter two being important mechanisms for horizontal gene transfer (Fig. 2A, B and Supplementary Fig. 6C, D)^15^. Therefore, PCS are equipped with functions associated with energy metabolism that benefit both the host and interactions with other PCS microbes, whereas TCS are equipped with functions that hijack or disturb host or neighboring commensal microbes.

As for the key differences in the functional and phenotypic differences between PCS and TCS, we hypothesized that PCS and TCS may differ in their growth rates in the host niche, the former outgrowing the latter. We tested this hypothesis in three ways. First, we estimated species growth rates from metagenomic samples by Growth Rate InDex (GRiD) analysis^16^ (Methods). For this, we stratified individuals into groups, enriched in PCS or TCS and found that in both groups GRiD scores of PCS were higher than TCS; they were the highest among PCS-enriched group (Fig. 2C, D). Second, we assessed species growth rates in bioreactors inoculated with healthy human stool samples, via GRiD analysis (Fig. 2E, F, Methods). We observed that the growth of PCS increased significantly over 24 h, whereas that of TCS did not change, demonstrating that PCS could outgrow the TCS. Third, we used genome-scale metabolic modeling (GEM) to simulate species growth rates^17–20^ on four different common diets (high fiber and high protein for plant- and animal-based diets) for 34 or 30 highly prevalent TCS and PCS, respectively (see Methods). The predicted growth rates of the selected PCS were significantly higher than of TCS (Fig. 2G, H and Supplementary Table 5). Furthermore, reaction essentiality analysis indicated that the GEMs of TCS were significantly more dependent on the substrate, often displaying amino acid auxotrophy (Supplementary Figs. 7 and 8). We hypothesize that the differences in growth rates and substrate dependence between PCS and TCS could underlie the directionality of the gut microbiome dynamics we report.

### Consistent observations of inflow and outflow probability in other independent longitudinal cohorts from different geographical regions

**Fig. 3:**
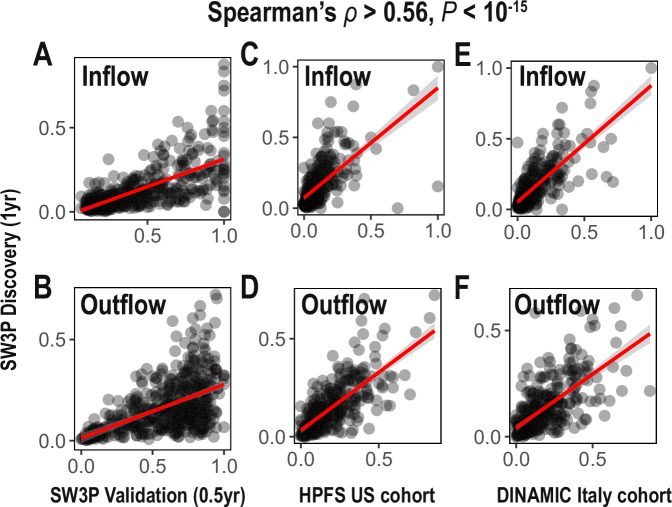
Consistent observations of TCS and PCS based on correlation of inflow/outflow probabilities in different sample times and cohorts across different countries. Inflow (**A**, **C**, **E**) and outflow (**B**, **D**, **F**) scores estimated from Swedish wellness (S3WP) discovery cohort of four visits were further validated by comparison with those from (**A**, **B**) two additional time points from the same S3WP cohort (validation cohort), **C**, **D** the American HPFS cohort and **E**, **F** the Italian DINAMIC cohort. We compared inflow scores and outflow scores between different datasets and found significant correlations with each other (Spearman’s correlation coefficients >0.56 and *p*-values < 10^−15^ for all comparisons).

To test whether the PCS and TCS assignments of species deduced from the analysis of the four time points in our discovery cohort persist over time, we collected and analyzed two additional time points with six months intervals from the 67 individuals of the same cohort (validation cohort) (Fig. 3A, B). Furthermore, to examine whether the assignments defined from a Swedish study are also found in other, geographically different regions, we analyzed two publicly available longitudinal cohorts, from Italy and USA^21,22^ (Fig. 3C–F). We generated the MGS profiles using the same gene catalog and downsizing threshold of 10 million reads for unbiased comparison. In all cases, for both TCS and PCS tendency (i.e., inflow and outflow) were significantly correlated with those found for the first four time points of our longitudinal cohort (Spearman’s correlation coefficients >0.56 for all comparisons). We conclude that PCS and TCS microbes are largely conserved and are thus a global feature of the human gut microbiome.

### Transient colonizing species are the biotic driver of the microbial dysbiosis in chronic diseases

**Fig. 4:**
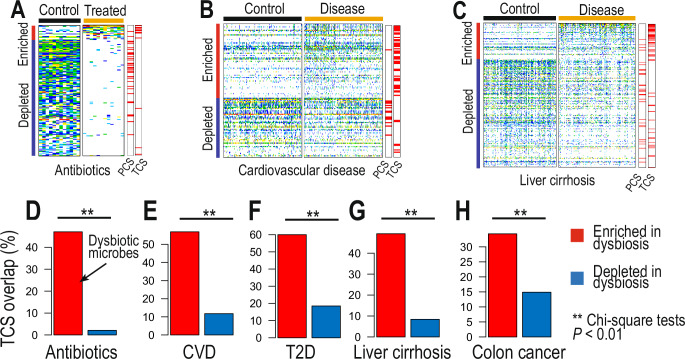
Dysbiotic microbes in acute and chronic conditions revealed as transient colonizing species. **A**–**C** Association of the acute and chronic conditions with PCS and TCS. Based on MGS abundance profiles (leftmost two boxes), the enriched or depleted MGSs were identified (underlined with red and blue colors on the left side of the boxes, respectively) in **A** antibiotics treatments (meropenem, gentamicin, and vancomycin), **B** cardiovascular disease (CVD), and **C** liver cirrhosis. Interestingly, TCS and PCS were significantly shared (rightmost two boxes) with enriched and depleted species in dysbiotic conditions, respectively, indicating strong associations of dysbiotic microbiome with the blooming of TCS microbes. **D**–**H** we showed significant TCS overlaps with enriched MGSs in dysbiotic conditions, compared to the depleted MGSs - **D** antibiotics, **E** cardiovascular disease, **F** type-2 diabetes, **G** liver cirrhosis, and **H** colon cancer (Chi-square tests *p*-values < 0.01).

In healthy individuals and stable conditions, it is likely that individuals’ gut microbiome would be populated with PCS and lacking in TCS, as we observed from representative physiological and functional properties, including growth rates and metabolic capacities. Therefore, we then questioned how PCS and TCS could shape the gut microbiome in dysbiotic conditions and drive the gut ecosystem to a low fitness composition. We first investigated acute changes in the gut microbiome driven by antibiotic treatment^3^. Based on shotgun metagenomics of 24 samples from 12 individual before antibiotic administration (meropenem, gentamicin, and vancomycin) and following 7 days administration, we identified enriched and depleted species (Fig. 4A) (Wilcoxon rank sum two-sided tests, *p*-values < 0.05). We found significant depletion of several species following antibiotic administration, leading to low diversity in antibiotic-treated individuals. Interestingly, we found that after 7 days of antibiotic treatment, host gut microbiomes were significantly enriched in TCS (hypergeometric test, *p*-value = 0.046), whereas there was a parallel depletion of 79% of PCS species in the same communities (hypergeometric test, *p*-value < 10^−15^) (Fig. 4A, D). Therefore, we identified that acute perturbations of the gut flora can significantly impact on the microbial flux of TCS microbes. We therefore hypothesize that the prolonged transition state of microbial flux in TCS microbes could be a basis for the initiation or transition to a dysbiotic state in chronic diseases.

To test this hypothesis, we profiled shotgun metagenomic cohorts of diseases associated with gut dysbiosis, including cardiovascular disease, type-2 diabetes, liver cirrhosis and colorectal cancer^14,23–25^ (Supplementary Table 6). Intriguingly, we found all the microbial species enriched in diseased conditions (Wilcoxon rank sum two-sided tests, *p*-values < 0.01) showed significant overlap with TCS microbes identified here (Chi-square tests, *p*-values < 0.01) (Fig. 4B, C, E–H). In summary, we found that all the enriched species in both acute and chronic conditions of dysbiosis were likely TCS, thus being potential initiators and drivers of disease pathogenesis (Fig. 4).

### Transient colonizing species were strongly associated with abnormal changes of host physiology

**Fig. 5:**
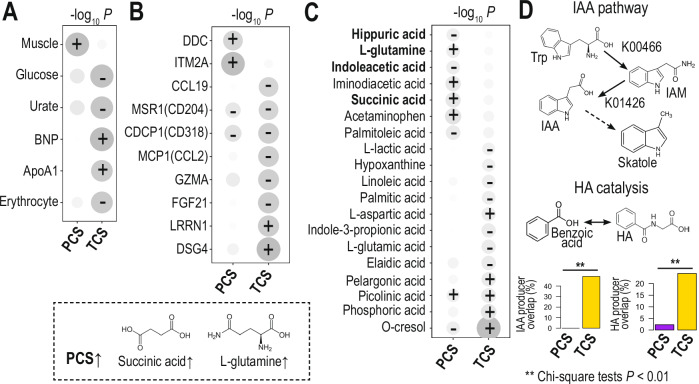
Persistent and transient colonizing species drives the changes of host physiology. We identified the associations of PCS/TCS abundance changes with (**A**) clinical chemistry and hematology variables for disease diagnosis, (**B**) serum metabolomics, and (**C**) serum proteomics. **A**–**C** PCS/TCS abundances were significantly associated with clinical and hematological variables, metabolomics, and proteomics by linear mixed-effects models (*p*-value < 0.05). Significant positive (+) and negative associations (−) are marked on a heatmap (size proportional to significance). Interestingly, TCS microbes were associated with increasing toxic compounds, including indole-acetic acid (IAA), and deregulated immune functions. Metabolites with bold names were key metabolites associated with PCS/TCS, which were shown with their chemical structure and pathways in **C** and **D**. Metabolites with bold names were key metabolites associated with PCS/TCS, which were shown with their chemical structure and pathways in **C** and **D**. **D** We identified MGSs equipped with IAA and HA biosynthetic pathways (i.e., containing KEGG ortholog terms, K00466 and K01426 or K01451). We found their significant overlaps with TCS (Chi-square tests *p*-value < 0.01).

As we observed the potential role of TCS enrichment in short term and long term dysbiotic gut microbial communities, we then questioned the potential implication of changes in TCS abundance on the physiological changes that could lead to disease pathology in dysbiotic conditions. To get a hint, we used previously generated serum proteomics and metabolomics data, together with clinical biochemistry and hematology data, from the same Swedish longitudinal cohort used earlier to identify the PCS/TCS clusters^26^. Here we performed an association study with PCS and TCS populations using linear mixed-effect models (Methods) (Fig. 5A–C and Supplementary Tables 7–9). First, we observed that PCS-enriched individuals were likely to have better exercise capacity (muscle mass), whereas those enriched TCS species were likely to have higher risks of heart failure (BNP marker), cardiovascular diseases (ApoA1), and immune disorders (Erythrocyte counts) (Fig. 5A and Supplementary Table 7). Next, we investigated serum proteomics associations with PCS and TCS population changes (Fig. 5B and Supplementary Table 8). In line with findings from the clinical biochemistry data set, we found that immune-related proteins, such as MSR1, CDCP1, MCP1, and GZMA, were decreased in individuals with higher TCS populations (Fig. 5B). Interestingly, individuals with higher PCS populations were enriched in ITM2A protein, a chondro-osteogenic differentiation marker^27,28^, as well as playing a role in T cell activation and myocyte differentiation^29,30^, thereby suggesting systemic changes in host physiology and immunity.

In addition, we investigated serum metabolites associated with changes of PCS and TCS populations (Fig. 5C and Supplementary Table 9). As expected from the functional and phenotypic analysis, the individuals with higher PCS populations were enriched with energy metabolites that fuel biosynthetic pathways, such as glutamine and succinic acid. These individuals also showed decreased levels of toxic compounds, such as hippuric acid (HA) and indoleacetic acid (IAA). Notably, these compounds have been predicted to penetrate the blood-brain barrier^31^ and affect neurological disorders^32^. In contrast, individuals with higher TCS populations were enriched in toxic compounds, including pelargonic acid (herbicide precursor), phosphoric acid (respiratory irritant), and O-cresol (uremic toxin). In addition, TCS species had metabolic pathways catalyzing toxic compounds more prevalently, such as HA and IAA, as compared to PCS species (Fig. 5D). Therefore, TCS microbes that have the ability to catalyze toxic compounds potentially producing systemic toxins, thereby driving and potentially even initiating disease pathology in multiple tissues.

## Discussion

Over the last 15 years, changes and shifts in the composition of the gut microbiome have been associated with an ever-increasing number of diseases at almost all body sites. These changes and shifts include everything from the dominance of an individual pathogenic species to loss of microbial diversity or substantive alterations in the species composition of these communities^33^. However, although different environmental factors that could influence these shifts have been identified, less is known about the kinetics and dynamics of these shifts. In this study, we have used longitudinal metagenomic data from healthy individuals to analyze the shifts in microbial communities, identifying species, events and microbial functions that potentially play a key role in movement between different community profiles.

Previous studies reported the temporal stability of gut microbiome composition in an individual^22,34,35^. Given the known fluctuations in gut microbiome composition resulting from environmental factors, this implies oscillations in species composition around an average value. The integrative analyses of temporal microbiome changes in a longitudinal study of healthy individuals that we have performed in this study have shown the existence of directionality in these compositional variations: species can be clustered into two populations with a tendency to either increase/maintain or decrease in abundance over time, termed persistent or transient colonizing species, respectively. As such, PCS-dominated microbiomes are associated with a stable microbial community with minimal changes, whilst TCS-dominated microbiomes are associated with changing communities that show little evidence of equilibrium. Given these features, microbial communities rich in TCS are likely to be either inherently unstable or in a state of transition between two different climax communities, whereas those rich in PCS are likely to be stable climax communities. This observation is in line with AKP that explains microbiome in normal condition tends to be resilient and perturbations can cause new equilibrium status, and likely leads to dysbiosis. Moreover, our findings could provide a distinguish between the key species drive the destabilization of the gut microbial community.

Importantly, transient colonizing species include most of the known gut-associated pathobionts, while persistent colonizing species are essentially devoid of these species. In line with this, the function-based analysis we carried out indicates that transient colonizing species potentially have a general negative impact on host physiology, as they have enriched accessory metabolism and secretion of virulence factors. Most interestingly, transient colonizing species tend to be enriched in different diseases while, in contrast, persistent colonizing species tend to be enriched in healthy individuals. We suggest that the tendency for the former to decrease and the latter to increase in healthy individuals is a previously unrecognized facet of the gut microbiome homeostasis.

The transient colonizing species tend to be facultative anaerobes with an oral origin (e.g., *Streptococcus* spp^36^.). This observation suggests that the high microbial flux seen in some dysbiotic communities and disease states is due to an increase in oral microbial transmission to the gut, possibly due to a decrease in gut microbiome resilience. This decreased resilience could be due to a number of different events, including sudden environmental changes or antibiotic treatments. Under these circumstances, the gut microbiome is repopulated with species transiting from the oral cavity. However, due to the significantly different environment in the gut, these species will have a lower competitive fitness than more normal gut tenants, meaning that their residency is transient in nature, unless other factors (i.e., host genetics/responses/treatments etc) have an impact. Notably, enrichment of oral species in the gut has been observed in several diseases^37–39^, and we suggest that increased mouth to gut microbial flow could be one of the global features of dysbiosis. However, it is also notable that some transient colonizers of gut origins also showed to be facultative anaerobic (e.g. *Enterococcus* spp.)^40^ and autotrophic (e.g. *Clostridium* spp.), which could be the reason on the lower rate of colonization in normal condition.

We have described the temporal dynamics of the gut microbiome through the discovery of transient colonizing and persistent colonizing species. The enrichment of persistent colonizing species in healthy populations is potentially due to their involvement in the degradation of storage carbohydrates, such as starch and fiber, which may account for their higher persistence. Similarly, these processes are also likely to lead to higher levels of short chain fatty acids and vitamins^41^, all of which have previously been shown to be of benefit to the host, providing both nutrition as well as regulating inflammation^42^. Thus, those communities dominated by PCS generally are commensalism, whilst those depleted in PCS and dominated by TCS are more generally associated with dysbiosis communities and diseases.

However, even though we associated PCS species with health benefits, abnormal increases of PCS species can be associated with disease pathogenesis. For examples, there were many short-chain fatty acids (SCFAs) and lactate-producers among PCS species, including *Ruthenibacterium lactatiformans*, but excessive production of lactate and SCFAs has been associated with many different diseases, including lactic acidosis, small intestinal bacterial overgrowth (SIBO) and neurodegenerative diseases like Parkinson’s diseases^43–45^. Therefore, investigation of PCS species with health benefits should be cautiously conducted.

In this study, we considered two mechanisms of TCS species enrichment in disease. First, the TCS species were enriched in competence mechanisms, facilitating import of genetic elements such as AMR, possibly conferring selective advantage in the gut through acquisition of genes via horizontal gene transfer. Enrichment of drug efflux mechanisms in TCS species might also confer resistance to antibiotics and other medications used in disease treatments, as well as to otherwise toxic metabolites. Further duplication of genes via these mechanisms would lead to overexpression of other genes, resulting in increases in otherwise tightly regulated factors or virulence genes. Second, the TCS species may plunder nutrients from the host, for example by utilizing simple monosaccharides to increase their abundance, and thereby depleting these metabolites for the host and other PCS microbes.

The significance of these findings in the human host can be seen when we look at the balance of PCS and TCS species in the diseased individual. The increased abundance of TCS species and their related metabolites are reflective of a more unstable microbiome that is potentially transitioning from a healthy to a diseased pathobiome. Whether the increase in TCS microbes represents a transitional state, or whether they represent a permanent pathological community is the key question. Given the lack of longitudinal data in diseased individuals, it is not possible to say from this study whether the increased numbers of TCS species is a transient event in a move to a new, dysbiotic but stable community, or whether it represents a new, unstable community itself. Given the association of disease-linked metabolites with TCS microbes, it is likely that a combination of the two is likely; the increasing abundance of TCS microbes likely represents an ongoing move from a eubiotic, balanced and healthy pathobiome to a progressively worsening, disease associated pathobiome. Thus, instead of looking for specific microbes or looking at a cross-sectional, blanket change in microbial diversity, this study indicates that by determining changes in the relative proportion of PCS/TCS microbes, it would be potentially possible to not only predict disease, but also to track disease progression in chronic diseases, assessing the efficacy of treatments, and predicting the rate of progression or any potential deterioration in status. However, we need to carefully consider the possibility that many unknown microbes were not captured due to the limitations of gene catalog references, which can be determined in either PCS or TCS microbes in other cohorts. In addition, bioreactor fermentation experiments of PCS and TCS growths should be replicated for many donors, which would increase the understanding of inherent property of PCS and TCS species, such as growth and fermentation products. Otherwise, further in vitro experiments of growth rates of specific PCS and TCS species needs to be carefully performed to elucidate their differential characters in growths. We also should note that there could be possibilities that passenger bacteria, that are not among the TCS or PCS, and can be associated with disease pathogenesis when they are depleted and requires more in-depth investigation.

## Methods

### Swedish wellness study population, sample collection, extraction, library prep and sequencing

The Swedish wellness study (S3WP) is an ongoing prospective cohort study based on the Swedish CArdioPulmonary bioImage Study (SCAPIS) with 30,154 individuals enrolled at ages between 50 and 64 years recruited from random sampling of the general Swedish population. A total of 86 healthy individuals were recruited in the study and followed longitudinally for two years. Examinations in SCAPIS include imaging to assess coronary and carotid atherosclerosis, clinical chemistry, anthropometry, and extensive questionnaires, as previously described^46^. All participants provided written informed consent. The study protocol conforms to the ethical guidelines of the 1975 Declaration of Helsinki.

For the time points 5 and 6 of microbiome data for the Swedish Wellness study, samples from 67 individuals were collected. Total genomic DNA was extracted with the MagPure Stool DNA LQ kit from Magen Biotechnology Co, Ltd. To each tube were 600 ul ATP/PVP, 600 ul PCI and MagPure bead added. The samples were bead beated in a FastPrep 96 at 1600 rpm for 1 min. The samples were incubated at 65 °C for 20 min and thereafter centrifuged for 3 min at 14,000 × *g*. 340 ul of the upper phase of each sample were transferred to a deep-well plate and placed in a SP960. The following reagent plate were prepared and placed in a SP960, RNaseA 10 ul /well (15 mg/ml), Reagent mix 640 ul/well (MagPure Particles N 30 ul, Proteinase K (20 mg/ml) 20 ul and Buffer MLE 590 ul), GW1 (650 ul/well), 75% Ethanol (1,1 ml/well) and EB buffer (100 ul/well). Sequencing was performed by the MGI DNBSEQ-T7 and the MGI DNBSEQ-G400 and checked sequencing depths (>10 million reads) for further metagenome analysis.

### Quality control/normalization of gene counts and species abundance profiling

We filtered out human reads and then mapped metagenomic data on the human gut gene IGC2 catalog by using the METEOR suite^47^. Based on the aligned reads, we estimated the abundance of each reference gene of the catalog, normalizing multiple mapped reads by their numbers and summing up normalized counts for a given gene. Reducing the variability by sequencing depths, gene count values were downsized into 10 million reads per sample; and any samples less than 10 million mapped reads were excluded from our dataset. Normalized gene counts were used for the quantification of metagenomic species (MGS) abundance using IGC2 defined species^48^ (https://data.inrae.fr/dataset.xhtml?persistentId=doi:10.15454/FLANUP) and R *momr* (MetaOMineR) package^49^. MGS abundances were estimated by the mean abundance of their 100 ‘marker’ genes (that is, the genes that correlate the most altogether). If less than 10% of the ‘marker’ genes were seen in a sample, the abundance of the MSPs was set to 0.

### Functional annotations of the human gut microbiome gene catalog

Based on *blastp* alignment (e-value = 10^−5^), the IGC2 catalog was annotated for the Antibiotic Resistant Determinants (ARD) of Mustard database (v1.0) (http://www.mgps.eu/Mustard/)^50^. Carbohydrate-active enzymes (CAZymes) family of the IGC2 catalog was annotated with Hidden Markov Models (HMMs) built from each CAZy family^51^, following a procedure previously described for other metagenomics analysis^52^. KEGG orthology terms of IGC2 proteins were annotated using *Diamond*^53^ against KEGG database (version 82). Virulent proteins of PATRIC^54,55^ were annotated with *blastp* against IGC2^11^. Phenotypes of MGS were manually checked based on JGI-GOLD phenotype of annotated taxa (organism metadata)^56^. Standalone anti-SMASH program (ver. 5) was used to identify biosynthetic gene clusters (BGCs) of MGSs^57^.

### Modeling temporal changes of normal gut microbiota during a year

First, we chose samples with sequential visits of given subjects and counted the presence/absence of all detected MGSs. To decide the detection limit here, we fitted all non-zero abundance of MGSs into gamma distribution after per-million scaling and log2-transformation using R *fitdistrplus* package. Based on estimated shape and rate parameters from fitted gamma distribution, we counted species presence only when its abundance exceeded a percentile (>1%) based on the gamma distribution. Presence/absence profiles were fitted into a two-state Markov chain model (i.e. states of presence and absence) to estimate state transition probabilities between presence and absence (R *markovchain* package). We did not include species of 0% and 100% prevalence (i.e., *Blautia wexlerae*, msp_0076) to Markov chain model. Here we estimated inflow probability of state transition from absence to presence, and outflow probability of state transition from presence to absence. For the estimation of species-retaining probabilities, we modeled presence/absence profiles as “events” and estimated the retaining probability from the survival rates of Kaplan–Meier estimates using R *survival* and *survminer* packages.

For the validation of inflow and outflow from the same Swedish wellness cohort, we additionally followed two supplementary visits (by every three months) and processed metagenomics data of 67 subjects (134 samples) after excluding subjects with either missing visits or low sequencing depth (less than 10 million mapped reads). For the validation of inflow and outflow from independent cohorts, we processed metagenomics data from Italy (DINAMIC cohort PRJEB33500)^58^ and USA (HPFS cohort PRJNA354235)^22^ after excluding subjects with missing visits or low sequencing depth. In HPFS cohort, we only took six-months interval samples of individuals, excluding one-day interval samples. We counted the presence/absence of MGSs from the abundance profiles in a similar way of calculation in Swedish wellness cohort, and calculated state transition probabilities between presence and absence (i.e., inflow and outflow) after fitting presence/absence profiles into a two-state Markov chain model.

### Transient and Persistent Colonizing Species (PCS and TCS) definition

Based on estimated inflow and outflow probabilities, we identified persistent colonizing species (PCS) (*P*_*inflow*_ > 0.3, and *P*_*outflow*_ < 0.3) and transient colonizing species (TCS) (*P*_*outflow*_ > 0.3 and *P*_*inflow*_ < 0.3) and calculated scaled abundance of PCS (*Z*_*PCS*_) and TCS (*Z*_*TCS*_) like below (1, 2).

1 $${z}_{{ij}}=\frac{{A}_{{ij}}-{{\rm{\mu }}}_{i}}{{\sigma }_{i}}$$

2 $${Z}_{{PCS}}\,{or}\,{Z}_{{TCS}}(j)=\frac{1}{\sqrt{n}}\mathop{\sum }\limits_{i}^{n}{z}_{{ij}}$$

where *i* is a given MGS belonging to PCS or TCS, *A*_*i*_ is the abundance of species *i*, *u*_*i*_ is mean abundance of species *i* over all wellness cohort samples (344 samples), *σ*_*i*_ is the standard deviation of species *i* over all wellness cohort samples, *j* is a given sample of wellness cohort, and *n* is the total number of PCS or TCS. Based on scaled abundance of single MGS (*z*_*ij*_), we calculated the aggregated z-score of all PCS species and TCS species (*Z*_*PCS*_ and *Z*_*TCS*_, respectively) by summing scaled MGS abundances for *n* species, where *Z*_*PCS*_ and *Z*_*TCS*_ follows standard normal distribution, independent of *n* value^59^.

### Microbial functions associated with persistent or transient colonizing species

Inflow/outflow scores of MGSs were tested for their associations with function/phenotype annotations of given MGSs (i.e., presence/absence of functions) using univariate linear regressions to identify PCS-enriched functions (i.e., functions enriched according to inflow scores of given MGS) or TCS-enriched functions (i.e., functions enriched according to outflow scores of given MGS). Significant associations of microbial functions to inflow/outflow scores were selected using adjusted *p*-values of predictor variables (i.e., microbial functions) <10^−3^ and regression coefficients >0.

### Associations between MGS abundance profiles and clinical metadata, proteomics and metabolomics

Scaled abundance of PCS and TCS species populations together (*Z*_*PCS*_ and *Z*_*TCS*_, respectively) were tested for their associations with clinical parameters, proteomics, and metabolomics considering random effects of individuals by linear mixed-effect models using R *lme4* packages (*p*-values < 0.05) like below (3):

3 $${Y}_{i}={Z}_{{PCS}}{\beta }_{{PCS}}+{Z}_{{TCS}}{\beta }_{{TCS}}+{u}_{i}+\epsilon$$

where *Y* is clinical parameter, protein or metabolite, *β*_*PCS*_ and *β*_*TCS*_ are coefficients of fixed effect variables, *Z*_*PCS*_ and *Z*_*TCS*_, respectively, *u*_*i*_ is a random intercept for subject *i*, and *ϵ* is residual.

In addition, we tested associations of single MGS with clinical parameters, proteins or metabolites of given samples of wellness cohorts by linear mixed-effect models like below (4):

4 $${Y}_{{ij}}={A}_{i}{\beta }_{i}+{u}_{j}+\epsilon ,i\in {\rm{PCS\; or\; TCS}},{A}_{i}={\rm{species\; abundance}}$$

where *Y* is clinical parameter, protein or metabolite, *β*_*i*_ is coefficient of fixed effect variable, *A*_*i*_, *u*_*j*_ is a random intercept for subject *j*, and *ϵ* is residual. We identified significant associations between MGS abundance and clinical parameters, proteins or metabolites, based on explained variance of fixed effect calculated using R *MuMIn* package (explained variance >10%).

### Faecal fermentation in ARCOL bioreactor

M-ARCOL is a one-stage fermentation system run under semi-continuous conditions that simulates the main physicochemical and microbial conditions encountered in the human colonic ecosystem^60^. It consists of pH and temperature controlled, stirred (400 rpm), airtight glass vessels inoculated with faecal samples from human volunteers and maintained under anaerobic conditions by the sole activity of resident microbiota. The set-up in this study consisted in a main bioreactor containing the luminal-associated microbiota and a connected glass compartment with mucin beads to simulate the mucus-associated microbiota. The system was operated to simulate the colonic conditions of healthy human adults as described earlier (temperature 37 °C, pH 6.3, retention time 24 h)^60,61^. The experiments were conducted in duplicate with faecal samples from two donors (one male and one female, ranging in age from 24 to 50 years, with no history of antibiotic or probiotic treatment 3 months prior the beginning of the study)^60^. Following faecal inoculation of the bioreactor, fermentations were conducted for a total duration of 9 days, including 1 day under fed batch and the following 8 days under semi-continuous mode. Samples were collected daily in the bioreactor^60,61^.

### In situ metagenomic measurement of growth rate by Growth Rate Index (GRiD) scores

The GRiD software (v1.3)^16^ was used to calculate the growth rate index from the metagenomic samples from Swedish wellness cohort and fecal samples inoculated into bioreactor and fermented for 24 h. Briefly, this software calculates a proxy of growth rate by mapping the metagenomics reads to microbial genomes and measuring the coverage ratio between the origin and terminus of replication. Since GRiD is sensitive to the representativeness and quality of the genome used, we created a GRiD custom database representative to the gut microbiota, using only high-quality draft genomes from the MGnify database^62^. First, we matched the MGS gene clusters to the MGnify genomes using a BLASTN procedure, with a 95% identity threshold. Then we kept only the MGnify genomes passing these criteria: (1) ≥95% gene completion, ≤5% contamination, (3) ≤100 contigs. This resulted in a GRiD database of 36 PCS microbial genomes (92% of all PCS species) and 194 TCS microbial genomes (38% of all TCS species). Finally, the GRiD growth rate values were considered only when: (1) the genome displayed at least 1X coverage in the metagenome (using the –c 1.0 parameter), (2) the genome displayed a species heterogeneity less than 0.3 (as recommended by the authors), in order to remove spurious growth rate index^16,62^.

### Reconstruction of Genome Scale metabolic Model (GEM) and constrained based modeling for inflow/outflow MGSs

We reconstructed the GEMs of 30 PCS species and 34 TCS species with high prevalence (≥10%) and taxonomy annotated at species-level (i.e., excluding unclassified MGSs) using the KEGG orthology (KO) annotation of the gut catalog. The KO profile of each MGS were mapped into KBase metabolic model^63^ as reference model to provide reaction profiles. Regarding the reaction profiles the context specific GEMs were reconstructed and the functionality of the models was checked based on the provided biomass objective function and the gap filling was done using the COBRA toolbox and the reference model. To investigate the response of the PCS and TCS microbes to environmental changes and calculate the perturbations, we used four different diets i.e., high protein- and fiber- plant based diets and high-protein and fiber omnivorous diets. The composition of the diet was converted to mmol/gDW*hour for the simulation in anaerobic situation and the growth rate for each model were predicted for each diet using constraint-based modeling. To check the dependence of the PCS and TCS species to the compounds as input or medium and autotrophy, we performed an essentiality analysis in which the inability of each MGS to synthesize the metabolites was simulated by closing the corresponding exchange reactions; decreased growth rate shows the dependence of the MGS to the metabolites for growth.

### Reporting summary

Further information on research design is available in the Nature Research Reporting Summary linked to this article.

## Supplementary information

Supplemental Figures and Supplemental Table legends

Reporting summary

Supplemental Tables

## Data Availability

The additional two time points for the 67 individuals from the Swedish wellness cohort gut metagenome data sequenced for this study can be found from the European Nucleotide Archive under the study accession PRJEB52380. The rest of datasets accession codes used in this study are available in Supplementary Table 6.

## Code availability

The R package used to perform modeling temporal changes of microbiome for inflow and outflow analysis together can be found at our GitHub repository link: https://github.com/sysbiomelab/mPackage and for the metagenomics analysis the pipeline can be found at https://github.com/sysbiomelab/meteor_pipeline.
