# Supplementary material for: Transient colonizing microbes promote gut dysbiosis and functional impairment
Source: NPJ Biofilms Microbiomes. 2024 Sep 8;10:80. doi: 10.1038/s41522-024-00561-1 (PMC11381545; doi:10.1038/s41522-024-00561-1)
Supplement: Supplementary file 1 — Supplemental Figures and Supplemental Table legends [file 41522_2024_561_MOESM1_ESM.pdf]

Transient colonizing microbes promote gut dysbiosis and functional impairment

Sunjae Lee[1,2], Victoria Meslier[3], Gholamreza Bidkhori[1], Fernando Garcia-Guevara[1,4], Lucie Etienne-Mesmin[5], Frederick Clasen[1], Junseok Park[6], Florian Plaza Oñate[3], Haizhuang Cai[1], Emmanuelle Le Chatelier[3], Nicolas Pons[3], Marcela Pereira[7], Maike Seifert[7], Fredrik Boulund[7], Lars Engstrand[7], Doheon Lee[6], Gordon Proctor[1], Adil Mardinoglu[1,4], Stéphanie Blanquet-Diot[5], David Moyes[1], Mathieu Almeida[3], S Dusko Ehrlich[3], Mathias Uhlen[4], Saeed Shoaie[1,4,*]

[1] Centre for Host-Microbiome Interactions, Faculty of Dentistry, Oral & Craniofacial Sciences, King's College London, SE1 9RT, UK

[2] School of Life Sciences, Gwangju Institute of Science and Technology, Gwangju, Republic of Korea, 61005

[3] University Paris-Saclay, INRAE, MetaGenoPolis, 78350 Jouy-en-Josas, France

[4] Science for Life Laboratory, KTH – Royal Institute of Technology, Stockholm, SE-171 21, Sweden

[5] Université Clermont Auvergne, INRAE, UMR 454 MEDIS, 28 place Henri Dunant, F-63000, Clermont-Ferrand, France

[6] Department of Bio and Brain Engineering, KAIST, 291 Daehak-ro, Yuseong-gu, Daejeon 305-701, Republic of Korea

[7] Centre for Translational Microbiome Research, Department of Microbiology, Tumour and Cell Biology, Karolinska Institutet, Stockholm, SE-171 77, Sweden

*Corresponding author: saeed.shoaie@kcl.ac.uk

Supplemental Figures and Supplemental Table legends

1 Supplementary Figures

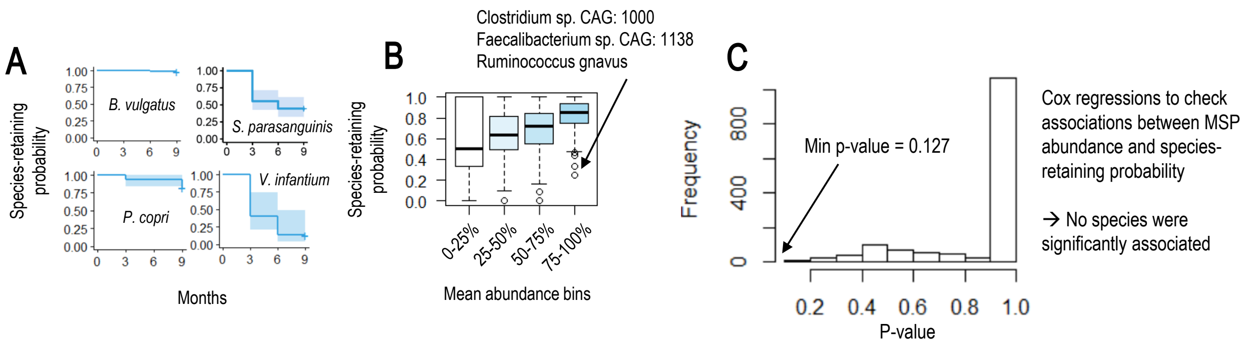

Supplementary Figure 1. Estimation of species-retaining probability from Swedish wellness longitudinal cohorts. A, species-retaining probability of representative species, such as *Bacteroides vulgatus, Prevotella copri, Streptococcus parasanguinis*, and *Veillonella infantium.* **B**, groups of species with different mean abundances (mean abundance bins) and corresponding species retention probabilities. We checked species retention probabilities according to mean abundance changes and found increasing trends, but with high variations. C, Cox regressions of MGS abundances to species-retention. No MGSs were significantly associated (all p-values <0.05).

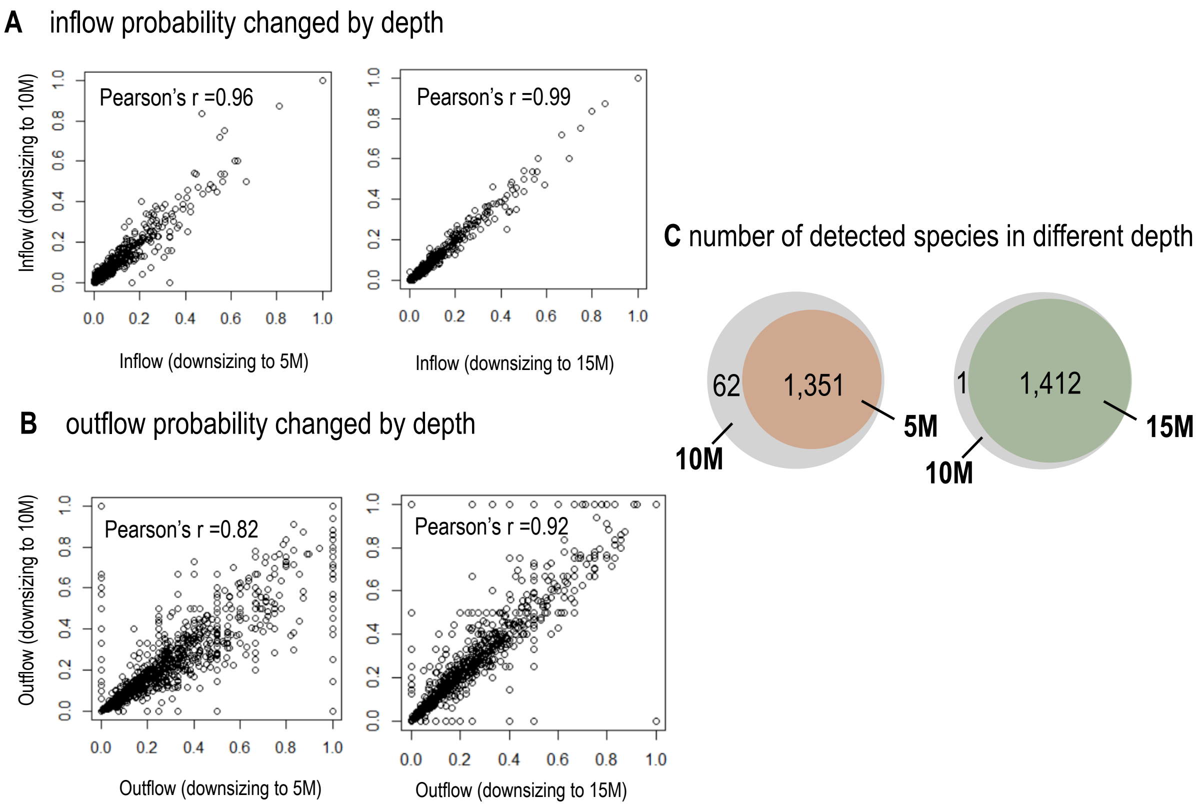

Supplementary Figure 2. Estimation of inflow, outflow probability according to sequencing depth. We checked the probability of **(A)** inflow, and **(B)** outflow changes for different downsizing levels, 5M, 10M, and 15M high quality reads. Inflow and outflow probability were highly correlated across different sequencing depths (Pearson's correlation coefficients >0.82, p-values $<10^{-15}$). **C**, in the scatter plots **(A)** and **(B)**, the undetected species at 5M reads and 10M reads downsizing were excluded. Venn diagram showed that there were 62 species missing at 5M reads downsizing compared to 10M reads, and 1 species missing at 15M reads compared to 10M reads.

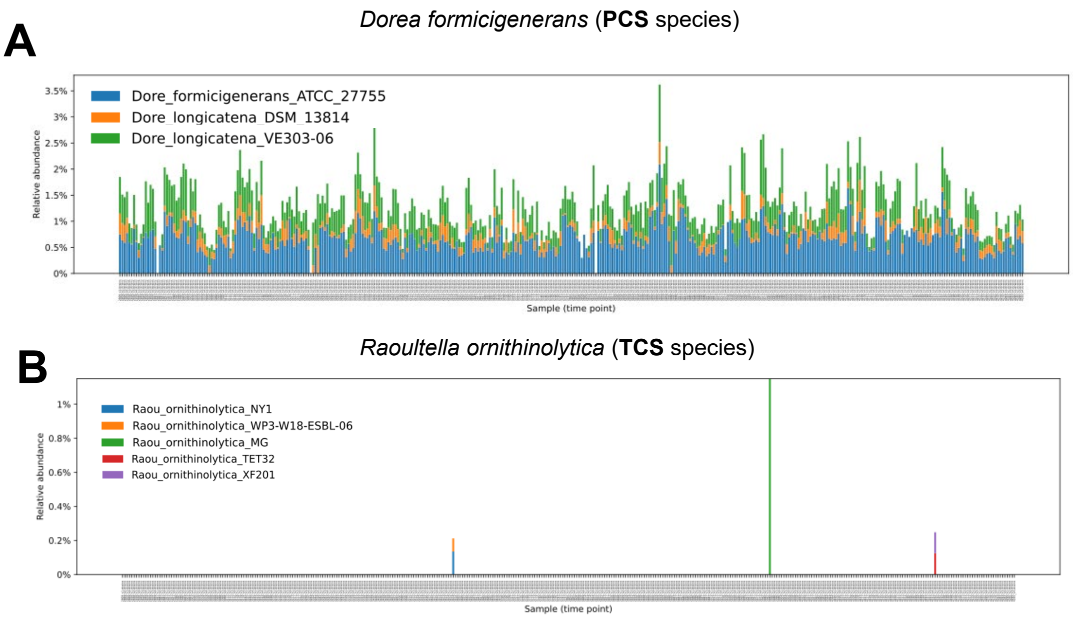

Supplementary Figure 3. Strain profiles of Swedish wellness cohort by StrainGE. . Using known strain genome profiles, we estimated relative abundance profiles of most closely related strains of representative species: **(A)** *Dorea formicigenerans*, PCS and **(B)** *Raoultella ornithinolytica*, TCS.

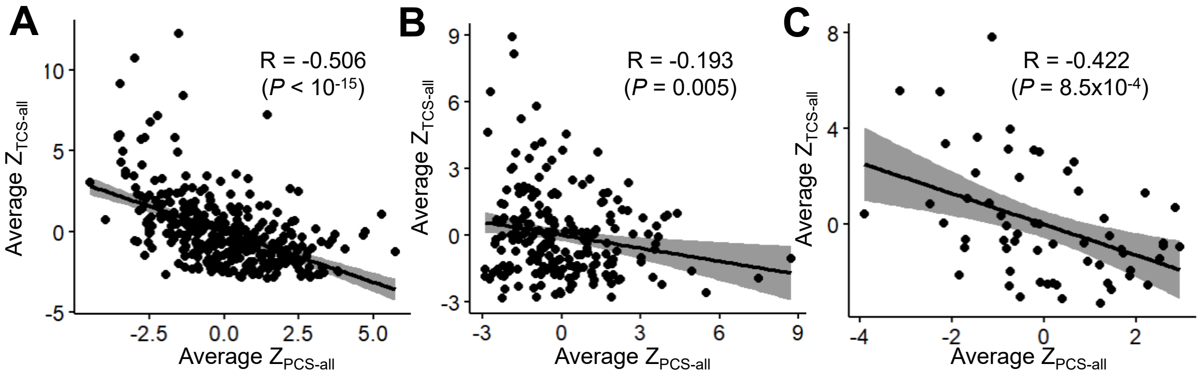

Supplementary Figure 4. Negative correlations between total PCS and TCS total abundances from cross-sectional datasets. We calculated Pearson's correlation coefficients between total scaled abundances of PCS and TCS abundances from **(A)** present Swedish cohort, **(B)** UK twin cohort, and **(C)** Germany colorectal cancer cohort

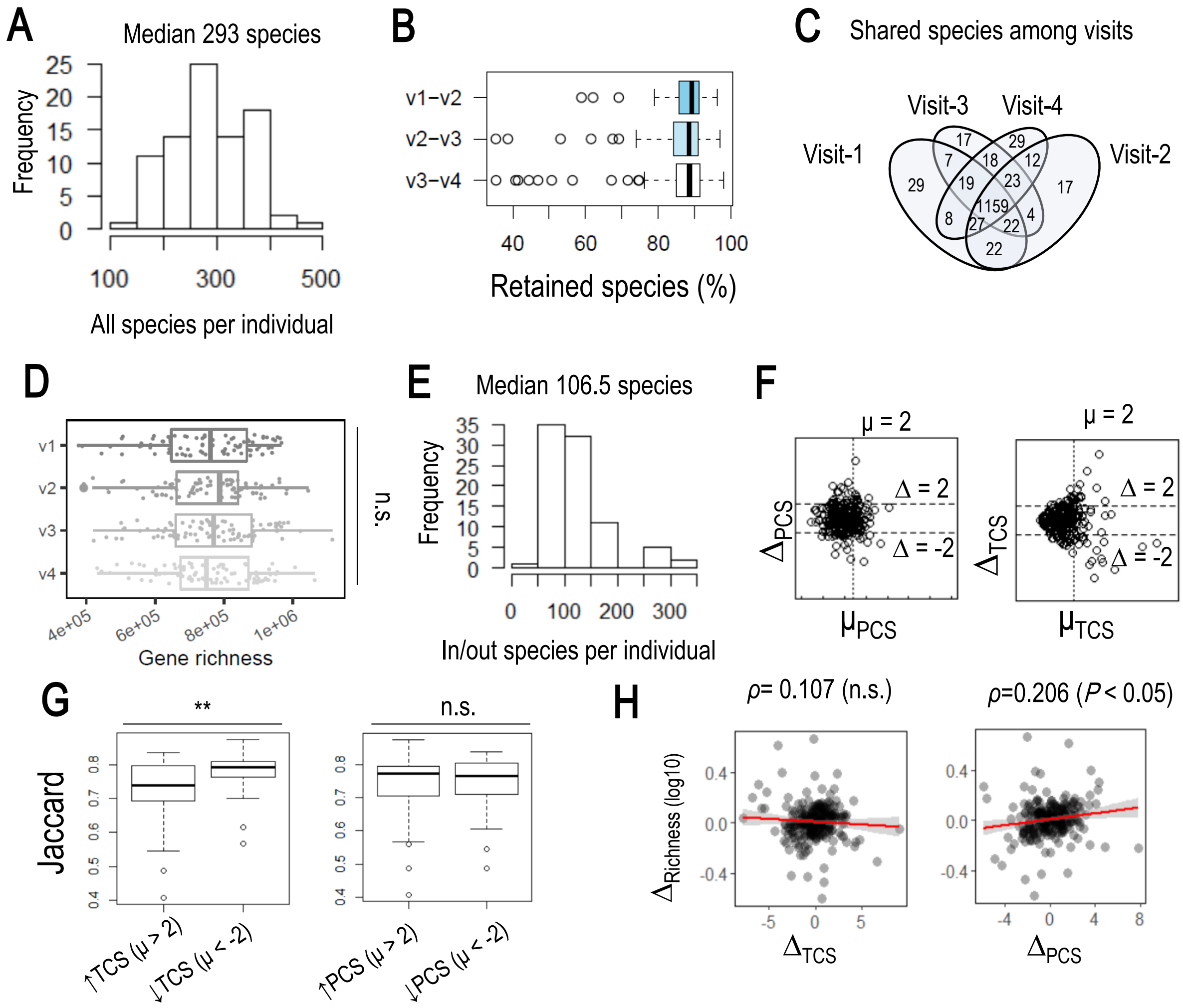
A
Median 293 species
Frequency
All species per individual
B
v1−v2
v2−v3
v3−v4
Retained species (%)
C
Shared species among visits
Visit-1
Visit-2
Visit-3
Visit-4
29
17
7
18
12
19
23
1159
8
27
22
4
22
17
29
D
v1
v2
v3
v4
n.s.
Gene richness
E
Median 106.5 species
Frequency
In/out species per individual
F
μ = 2
Δ = 2
Δ = -2
Δ_{PCS}
μ_{PCS}
Δ_{TCS}
μ_{TCS}
G
Jaccard
**
n.s.
↑TCS (μ > 2)
↓TCS (μ < -2)
↑PCS (μ > 2)
↓PCS (μ < -2)
H
ρ= 0.107 (n.s.)
ρ=0.206 (P < 0.05)
$\Delta_{Richness}$ (log10)
Δ_{TCS}
Δ_{PCS}

Supplementary Figure 5. Temporal dynamics of individual gut microbiome composition. A, the frequency of identified MGSs per individual. The median number of MGSs identified per individual for 86 individuals across quarterly visits had was 293. **B**, fraction of retained species between sequential visits. **C**, overlap of species identified by visits. Most species (1,159 MGSs) were shared among visits. **D**, gene richness of 86 individuals by visit. We examined the microbial diversity of 86 individuals by gene richness, counting the detected microbial genes of the reference catalogue per sample after downsizing mapped reads to 10 million reads. Based on t-tests, we found that there were no significant differences in gene richness among different visits of 86 individuals. **E**, species appearing or disappearing between visits. A median of 106.5 species appeared or disappeared per individual at the next visit. **F**, abundance changes of persistent colonizing species (PCS) and transient colonizing species (TCS) between visits (Δ PCS and Δ TCS, respectively) according to mean values of visits (µPCS and µTCS, respectively). Unlike TCS, the abundance of PCS rarely changed between visits (Δ PCS <2) according to the increase in mean values (µPCS). **G**, intra-individual similarity of PCS-enriched individuals (right panel) and TCS-enriched individuals (left panel). We identified TCS-enriched individuals among 86 healthy individuals based on scaled total abundance of TCS species by Z-score (Zt and Zt+1) and its mean between visits (TCS $= \frac{1}{2} \times$ (Zt + Zt+1) >2). TCS-enriched individuals were less similar between visits than TCS-depleted individuals (student's t-test p-value <0.01), whereas PCS-enriched individuals were similar between visits, similar to PCS-depleted individuals. **H**, correlations of changes in gene richness to TCS, and PCS (left, and right, respectively). Changes in PCS abundance (ΔPCS) were significantly correlated with richness changes (Pearson's correlation test, p-value <0.05), whereas changes in BMI and TCS species (ΔBMI and ΔTCS, respectively) were not significant.

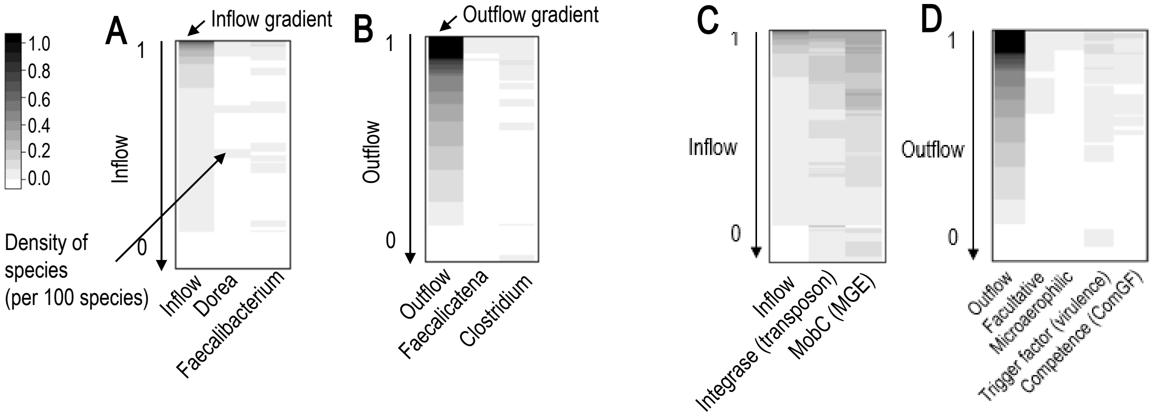

Supplementary Figure 6. Functional association with microbiome changes. A-D, inflow/outflow score-associated genera/functions (i.e., genera/functions enriched in PCS/TCS microbes). Based on univariate linear regressions, we found taxonomy **(A-B)** or functions **(C-D)** significantly enriched in MGSs with high inflow/outflow scores (linear regressions, adjusted p-value <0.01) and showed some of enriched genera and functions as examples in the heatmap. By decreasing inflow/outflow scores (leftmost column), we showed the density of species of given taxonomy or functions within sliding windows of 100 MGSs along MGSs sorted with given inflow/outflow scores (right columns). We found *Dorea* and *Faecalibacterium* spp. were significantly associated with increased inflow scores (linear regressions, adjusted p-values 5.0×10^{-4} and 1.2×10^{-3}, respectively), whereas *Faecalicatena* and *Clostridium* spp. significantly associated with increased outflow scores (linear regression, adjusted p-values = 0.0069 for both genera). In addition, integrase (transposon) and MobC (plasmid mobilization relaxosome) were significantly associated with increased inflow scores (linear regressions, adjusted p-values 3.2×10^{-27} and 6.5×10^{-15}), whereas facultative phenotype, microaerophilic phenotype, trigger factor (virulence) and ComGF (competence) associated with increased outflow scores (linear regressions, adjusted p-values 4.0×10^{-8}, 3.3×10^{-3}, 1.2×10^{-9}, and 1.4×10^{-5}, respectively).

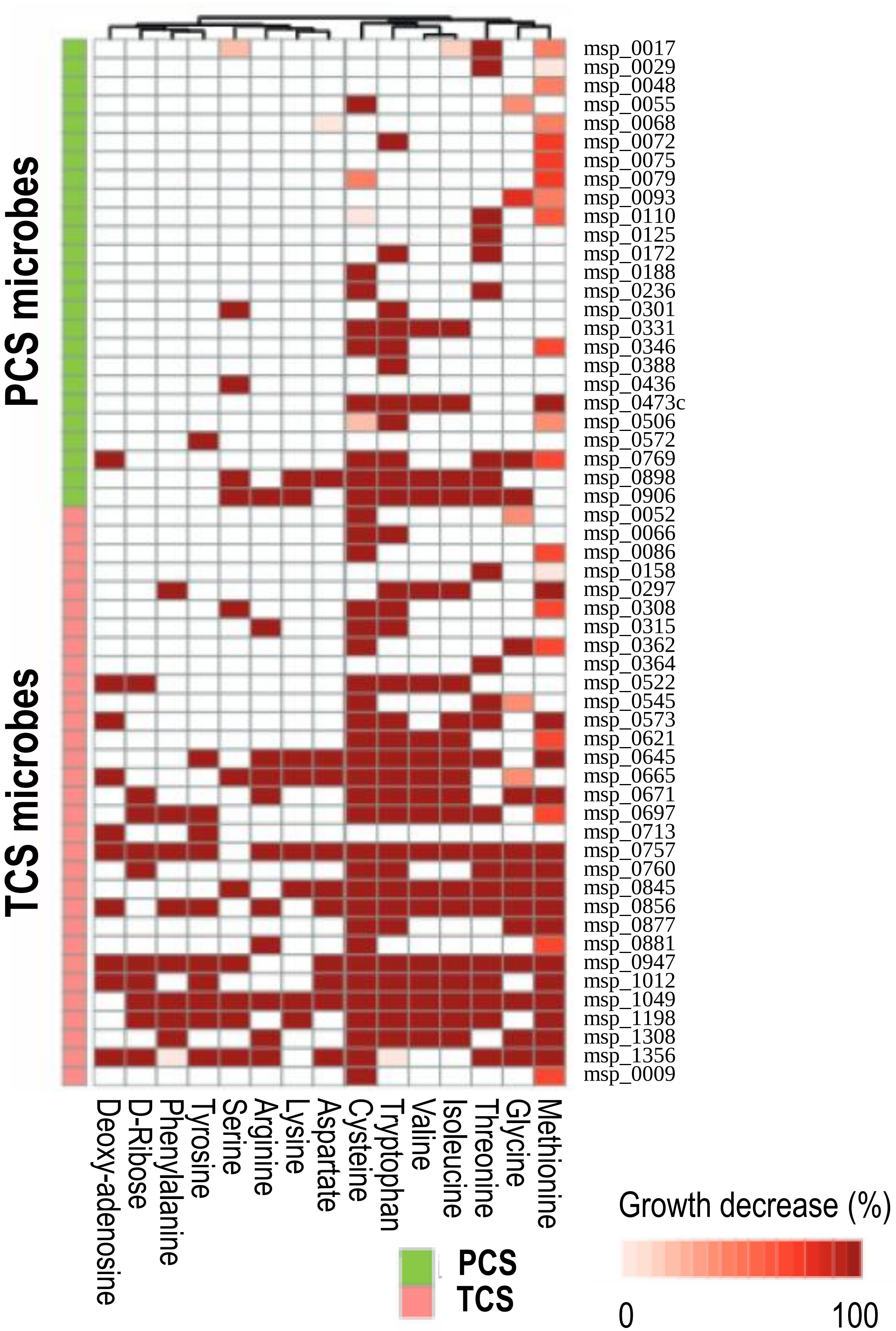

PCS microbes
TCS microbes
msp_0017
msp_0029
msp_0048
msp_0055
msp_0068
msp_0072
msp_0075
msp_0079
msp_0093
msp_0110
msp_0125
msp_0172
msp_0188
msp_0236
msp_0301
msp_0331
msp_0346
msp_0388
msp_0436
msp_0473c
msp_0506
msp_0572
msp_0769
msp_0898
msp_0906
msp_0052
msp_0066
msp_0086
msp_0158
msp_0297
msp_0308
msp_0315
msp_0362
msp_0364
msp_0522
msp_0545
msp_0573
msp_0621
msp_0645
msp_0665
msp_0671
msp_0697
msp_0713
msp_0757
msp_0760
msp_0845
msp_0856
msp_0877
msp_0881
msp_0947
msp_1012
msp_1049
msp_1198
msp_1308
msp_1356
msp_0009
Deoxy-adenosine
D-Ribose
Phenylalanine
Tyrosine
Serine
Arginine
Lysine
Aspartate
Cysteine
Tryptophan
Valine
Isoleucine
Threonine
Glycine
Methionine
PCS
TCS
Growth decrease (%)
0
100

Supplementary Figure 7. Essentiality analysis. The heatmap of metabolites leading to the decrease of growth rate or lethality in the PCS and TCS showed that essentiality is significantly different between the group (Fisher's exact test, p-value <0.05).

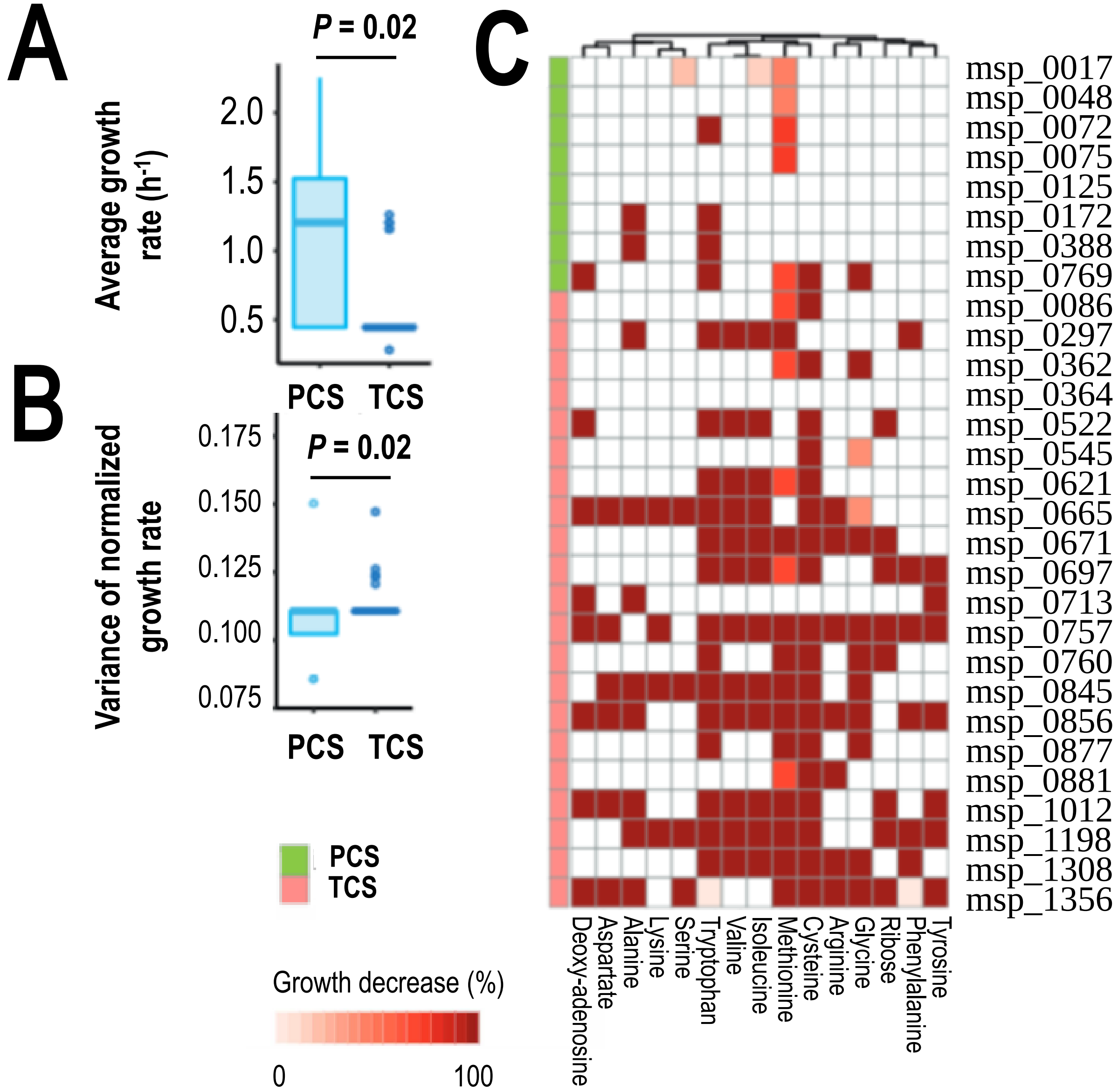

Supplementary Figure 8. Constraint-based modelling of common PCS and TCS detected in three different cohorts, Swedish SW3P discovery cohort, Italian DINAMIC, and American HPFS cohorts A, boxplot of growth rates of common PCS and TCS, predicted in the four different diets **B**, boxplot of variance of growth rate changes for four different diets. To compare the variance among the MGSs the growth rates were normalized. **C**, the heatmap of metabolites leading to decrease of growth rate or lethality in the common PCS and TCS by essentiality analysis showed that essentiality is significantly different between the group (Fisher's exact test, p-value <0.05).

2 Supplementary Table Legends

Supplementary Table 1. Demographics of Swedish wellness cohorts of 86 individuals.

Supplementary Table 2. Summary of Markov chain model (MCM) statistics and species-retaining probability.

Supplementary Table 3. statistics of associations between inflow/outflow scores and microbial functions.

Supplementary Table 4. functional classes enriched in PCS and TCS microbes, based on respective associations with inflow and outflow scores

Supplementary Table 5. Summary of the simulated growth rates for 34 TCS and 30 PCS GEMs based on four different dietary inputs.

Supplementary Table 6. Description of public shotgun metagenomic datasets used in this study.

Supplementary Table 7. statistics of association between PCS/TCS populations and clinical parameters.

Supplementary Table 8. statistics of association between PCS/TCS populations and proteomics.

Supplementary Table 9. statistics of association between PCS/TCS populations and metabolomics.

Supplementary Table 10. MSP abundance table of wellness cohorts of four visits Supplementary Table 11. Average metadata (age, gender, BMI) for the wellness cohort.
