# Supplementary material for: Transient colonizing microbes promote gut dysbiosis and functional impairment
Source: NPJ Biofilms Microbiomes. 2024 Sep 8;10:80. doi: 10.1038/s41522-024-00561-1 (PMC11381545; doi:10.1038/s41522-024-00561-1)
Supplement: Supplementary file 2 — Reporting summary [file 41522_2024_561_MOESM2_ESM.pdf]

Corresponding author(s): Saeed Shoaie

Last updated by author(s): Mar 22, 2024

Reporting Summary

Nature Portfolio wishes to improve the reproducibility of the work that we publish. This form provides structure for consistency and transparency in reporting. For further information on Nature Portfolio policies, see our Editorial Policies and the Editorial Policy Checklist.

Statistics

For all statistical analyses, confirm that the following items are present in the figure legend, table legend, main text, or Methods section.

n/a	Confirmed	
☐	☒	The exact sample size (*n*) for each experimental group/condition, given as a discrete number and unit of measurement
☒	☐	A statement on whether measurements were taken from distinct samples or whether the same sample was measured repeatedly
☐	☒	The statistical test(s) used AND whether they are one- or two-sided<br>*Only common tests should be described solely by name; describe more complex techniques in the Methods section.*
☐	☒	A description of all covariates tested
☐	☒	A description of any assumptions or corrections, such as tests of normality and adjustment for multiple comparisons
☐	☒	A full description of the statistical parameters including central tendency (e.g. means) or other basic estimates (e.g. regression coefficient) AND variation (e.g. standard deviation) or associated estimates of uncertainty (e.g. confidence intervals)
☐	☒	For null hypothesis testing, the test statistic (e.g. *F*, *t*, *r*) with confidence intervals, effect sizes, degrees of freedom and *P* value noted<br>*Give P values as exact values whenever suitable.*
☒	☐	For Bayesian analysis, information on the choice of priors and Markov chain Monte Carlo settings
☒	☐	For hierarchical and complex designs, identification of the appropriate level for tests and full reporting of outcomes
☐	☒	Estimates of effect sizes (e.g. Cohen's *d*, Pearson's *r*), indicating how they were calculated

Our web collection on statistics for biologists contains articles on many of the points above.

Software and code

Policy information about availability of computer code

Data collection	Microbiome profile of wellness cohort from v1 to v4 is available in supplementary table. The additional two time points for the 67 individuals of wellness cohort gut metagenome data sequenced for this study can be found from the European Nucleotide Archive under the study accession PRJEB52380. The rest of publicly available datasets accession codes used in this study are available in Table S6.
Data analysis	Metagenome data was analyzed by meteor and R MOMR package. functional annotation was done by HMMER, Diamond, blastp and anti-smash. survival analysis was done by R survival and survminer packages. Growth rates of microbes from shotgun metagenome was done by GRiD software. linear-mixed effect model analysis was done by R lme4 and MuMIN packages.

For manuscripts utilizing custom algorithms or software that are central to the research but not yet described in published literature, software must be made available to editors and reviewers. We strongly encourage code deposition in a community repository (e.g. GitHub). See the Nature Portfolio guidelines for submitting code & software for further information.

Data

Policy information about availability of data

All manuscripts must include a data availability statement. This statement should provide the following information, where applicable:

- Accession codes, unique identifiers, or web links for publicly available datasets
- A description of any restrictions on data availability
- For clinical datasets or third party data, please ensure that the statement adheres to our policy

The additional two time points of 67 individuals from the wellness cohort gut metagenome data sequenced for this study can be found in the European Nucleotide Archive under the study accession PRJEB52380. The rest of datasets accession codes used in this study are available in Table S6.

Research involving human participants, their data, or biological material

Policy information about studies with human participants or human data. See also policy information about sex, gender (identity/presentation), and sexual orientation and race, ethnicity and racism.

Reporting on sex and gender	We did not perform sex or gender-based analysis in this study.
Reporting on race, ethnicity, or other socially relevant groupings	we did not report race, ethnicity or socially relevant groupings in this study.
Population characteristics	We provided demographic information in the supplementary table.
Recruitment	The Swedish wellness study (S3WP) is an ongoing prospective cohort study based on the Swedish CArdioPulmonary bioImage Study (SCAPIS) with 30,154 individuals enrolled at ages between 50 and 64 years recruited from random sampling of the general Swedish population. A total of 86 healthy individuals were recruited in the study and followed longitudinally for two years. Examinations in SCAPIS include imaging to assess coronary and carotid atherosclerosis, clinical chemistry, anthropometry, and extensive questionnaires, as previously described. (G. Bergström et al., Journal of Internal Medicine, 2015). All participants provided written informed consent. The study protocol conforms to the ethical guidelines of the 1975 Declaration of Helsinki.
Ethics oversight	SCAPIS has been approved as a multicentre trial by the ethics committee at Umeå University and adheres to the Declaration of Helsinki.

Note that full information on the approval of the study protocol must also be provided in the manuscript.

Field-specific reporting

Please select the one below that is the best fit for your research. If you are not sure, read the appropriate sections before making your selection.

☒ Life sciences ☐ Behavioural & social sciences ☐ Ecological, evolutionary & environmental sciences

For a reference copy of the document with all sections, see nature.com/documents/nr-reporting-summary-flat.pdf

Life sciences study design

All studies must disclose on these points even when the disclosure is negative.

Sample size	86 normal subjects recruited for the longitudinal gut microbiome analysis. for the time points 1 to 4, 86 samples per visit were available from the previously published study (https://doi.org/10.1016/j.chom.2022.03.002). For the time point 5 and 6 reported in this study, samples from 67 individuals were collected and analysed.
Data exclusions	n.a.
Replication	n.a.
Randomization	n.a.
Blinding	n.a.

Reporting for specific materials, systems and methods

We require information from authors about some types of materials, experimental systems and methods used in many studies. Here, indicate whether each material, system or method listed is relevant to your study. If you are not sure if a list item applies to your research, read the appropriate section before selecting a response.

Materials & experimental systems

n/a	Involved in the study
☒	☐ Antibodies
☒	☐ Eukaryotic cell lines
☒	☐ Palaeontology and archaeology
☒	☐ Animals and other organisms
☒	☐ Clinical data
☒	☐ Dual use research of concern
☒	☐ Plants

Methods

n/a	Involved in the study
☒	☐ ChIP-seq
☒	☐ Flow cytometry
☒	☐ MRI-based neuroimaging

Plants

Seed stocks	n.a.
Novel plant genotypes	n.a.
Authentication	n.a.
